# Early Biomarkers and Hearing Impairments in Patients with Neonatal Hypoxic–Ischemic Encephalopathy

**DOI:** 10.3390/diagnostics11112056

**Published:** 2021-11-06

**Authors:** Da-Yang Chen, Inn-Chi Lee, Xing-An Wang, Swee-Hee Wong

**Affiliations:** 1 Department of Pediatrics, Chung Shan Medical University Hospital, Taichung 40201, Taiwan; kazyyang@gmail.com (D.-Y.C.); a7710355@yahoo.com.tw (S.-H.W.); 2 Division of Pediatric Neurology, Department of Pediatrics, Chung Shan Medical University Hospital, Taichung 40201, Taiwan; 3 Institute of Medicine, School of Medicine, Chung Shan Medical University, Taichung 40201, Taiwan; 4 Division of Neonatology, Department of Pediatrics, Chung Shan Medical University Hospital, Taichung 40201, Taiwan; i0000528528@yahoo.com.tw

**Keywords:** newborn, HIE, biomarker, hearing impairment, neurodevelopment

## Abstract

Identifying biomarkers for hearing impairments (HIs) in patients with neonatal hypoxic–ischemic encephalopathy (HIE), to initialize early hearing habilitation, is crucial. Seventy-eight neonates with HIE were divided into the following two groups: those with HIs and those without HIs. We compared those patients with 11,837 newborns without HIE, and analyzed the risk factors of HIs among neonatal HIE. Of the 78 patients, 11 were confirmed to have an HI, which is a substantially higher percentage than in the 11,837 newborns without HIE (14.1% vs. 0.87%; *p* < 0.001). More patients with moderate-to-severe HIE had confirmed HIs (*p* = 0.020; odds ratio, 8.61) than those with mild HIE. Clinical staging, and blood lactate and glucose levels could be predictive factors for HIs among patients with HIE. The patients who exhibited HIs had significantly higher lactate (104.8 ± 51.0 vs. 71.4 ± 48.4; U = 181, *p* = 0.032) and serum glucose (159.5 ± 86.1 vs. 112.1 ± 62.3; U = 166, *p* = 0.036) levels than those without HIs. A higher prevalence of HIs was noted in the patients with stage III HIE than those with stage II HIE (43.8% vs. 10%; *p* = 0.008). The degree of HI correlated with brain anomalies and neurodevelopmental outcomes at 1 year of age. Clinical staging, and blood lactate and glucose levels could be predictive factors for HIs among patients with HIE.

## 1. Introduction

Hearing impairments (HIs) in newborns can cause a serious obstacle to their development in language, cognition, and education [1,2,3], and can have considerable effects on their future development if the HI is not managed [1]. The percentage of newborns with HIs has been estimated to be 0.5–5 out of every 1000 newborns and infants [1,2,3]. The development of an HI after birth can be caused by numerous etiologies, including congenital infection, a stay in the neonatal intensive care unit for more than 5 days, assisted ventilation, exposure to ototoxic medications or loop diuretics, hyperbilirubinemia that requires an exchange transfusion, craniofacial anomalies, neurodegenerative disorders, or meningitis [4]. Hypoxic–ischemic encephalopathy (HIE) is a common cause of neonatal death, neurodevelopmental delays, and HI.

Birth asphyxia is a physiological disorder that can develop in newborns as a result of a prolonged or profound disparity between oxygen demand and oxygen delivery [5]. Moderate-to-severe cases can cause irreversible cerebral cell damage and death, leading to a syndrome of HIE. HIE may lead to an altered conscious state, autonomic instability, absence of primitive reflexes, neonatal seizures, and death. Long-term consequences include epilepsy, intelligence disability, cerebral palsy, and hearing and visual impairment. A proportion of neonates with HIE will develop various degrees of HIs, despite the use of rescue hypothermia, which is standard care for those with moderate-to-severe neonatal HIE. 

Inducing hypothermia among those patients with neonatal HIE is effective, leads to few adverse effects among newborns [6,7,8], and can rescue the brain from parenchymal injury. Although therapeutic hypothermia is clinically used to reduce neurological injuries secondary to HIE, a 45–55% risk of death or moderate–severe disability in treated infants [6,7,9], including hearing damage, can occur. The causes of HIs are uncertain; however, hypoxic damage to the internal ear nuclei is probable. Despite the effectiveness of inducing hypothermia among newborns with moderate-to-severe neonatal HIE, the therapeutic effect is not clear among newborns with both HIE and HI. One concern was that hypothermia causes HIs, and the probable associated effects of therapeutic hypothermia are risks of intracranial hemorrhage and cardiopulmonary instability [6]; however, these correlations must be further clarified [10,11]. 

The risk factors of HIE with hearing loss are a history of receiving mechanical ventilation, use of ototoxic medications, admission to the neonatal intensive care unit for more than 7 days, and a low Apgar score [12]. However, the most crucial factor is neonatal HIE itself, which may cause hearing impairment. The common biomarkers for predicting the degree of neonatal HIE from the blood are lactate, lactate dehydrogenase (LDH), troponin T, creatine phosphokinase (CK), the urine ratio of lactate/creatinine (L/C), and troponin [13]. Absolute lactate values can be used as an auxiliary factor to make early estimates of long-term outcomes for newborns with neonatal asphyxia, who are being treated with therapeutic hypothermia [14]. A concentration of troponin T satisfactorily predicted mortality prior to discharge [15], and an elevated level of troponin I is a biomarker of myocardial ischemia among adults and children [16]. Therefore, we analyzed early blood biomarkers to predict HI. 

The early diagnosis of hearing problems and the rapid initialization of a hearing habilitation program are vital for a favorable long-term prognosis in neonatal neurodevelopment, particularly in language and cognition. As identifying the risk factors for HIs is also valuable for managing neonatal HIE, to avoid further damage, we investigated the risk factors of hearing damage in patients with neonatal HIE. We suggest early intervention for those patients with an HI.

## 2. Patients and Methods 

### 2.1. Patients with Neonatal HIE Were Enrolled 

**Figure 1:**
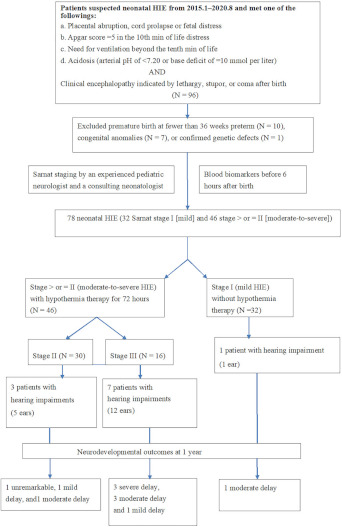
Flowchart of the study procedure for patients with neonatal hypoxic–ischemic encephalopathy and hearing impairments.

We retrospectively reviewed the records of patients with neonatal HIE at Chung Shan Medical University Hospital, located in central Taiwan, by identifying those with a clinical history of fetal distress, metabolic acidosis, or needing positive-pressure ventilation immediately after birth. HIE was classified by clinical Sarnat staging into stages I (mild), II (moderate), and III (severe) [6,7,17] by an experienced pediatric neurologist and consulting neonatologist. Overall, 96 patients had neonatal HIE, of which 32 patients had stage I, and 46 had stage II or III. Eighteen patients were excluded because they had congenital anomalies (N = 7), were preterm (N = 10), or had confirmed genetic defects (N = 1) (Figure 1). Further examinations of those with HIE included a complete blood count (CBC) and biochemistry, including blood gas, liver function (aspartate transaminase (GOT) and alanine transaminase (GPT)), cardiac enzymes (troponin I and creatine kinase-Mb by chemiluminescence immunoassays), creatine phosphokinase (CK), renal function (blood urea nitrogen (BUN) and creatinine), coagulation study (prothrombin time (PT) and activated partial thromboplastin time (aPTT)), lactate, lactic dehydrogenase (LDH), sodium (NA), potassium (K), glucose, and albumin. For moderate-to-severe HIE, magnetic resonance imaging (MRI) and hearing tests (automated auditory brainstem response (aABR) and auditory brainstem response (ABR)) [3] were conducted after hypothermia therapy and before discharge to evaluate the neurological consequence. 

### 2.2. Universal Neonatal Hearing Screening and Audiometric Measures 

From 2012 to 2020, we performed global newborn hearing screening for a total of 11,837 newborns. Newborn hearing screening was performed by aABR after birth for all newborns. For patients who failed the aABR twice, ABR, otoacoustic emissions (OAE), and steady-state evoked potentials (SSEP) were performed [3]. The ABR was adjusted by an experienced pediatric neurologist, or ear, nose, and throat doctor. The wavelengths were analyzed and the latency of peak V was defined. The degree of hearing loss was classified as normal (>25 and  ≤35 dB nHL), mild (>35 and  ≤45 dB nHL), moderate (>45 and  ≤65 dB nHL), severe (>65 and  ≤90 dB nHL), or profound (>90 dB nHL) [18,19].

### 2.3. Neurodevelopmental Outcomes Study

The neurodevelopmental outcomes were evaluated at 1 year of age. The Bayley Scales of Infant and Toddler Development, Third Edition (Bayley-III), were adopted to evaluate patients at the age of >1 year. The Bayley-III scores were interpreted as follows: normal, ≥85; mild, 70 ≤ and <85; moderate, ≤55 and <70; severe, <55 [20,21]. The cognitive and motor subscales of the Bayley-III scores were used to express neurodevelopmental outcomes. 

### 2.4. Statistical Analysis

Significant differences between the groups were evaluated using an independent *t*-test to compare the means of two independent groups, or a chi-square test between categorical variables. The Fisher exact test was used when sample sizes were small. The odds ratio (OR), the association between a variable and an outcome, was calculated by dividing the odds of the first group by the odds of the second group. If the sample distribution was nonparametric, a Mann–Whitney U test was performed. Significance was set at *p* < 0.05. All statistical tests were conducted using SPSS (version 14.0; SPSS Institute, Chicago, IL, USA).

Patient charts were retrospectively reviewed in all cases. Ethical approval of the study was provided by Chung Shan Medical University Hospital’s Internal Review Board (IRB #: CS2-14003), and the study was performed in accordance with relevant guidelines.

## 3. Results 

### 3.1. aABR Failure Rate in All Newborns Tested in Our Hospital and Neonatal HIE

The failure rate of neonatal hearing screening in newborns, from a total of 11,837 newborns in our hospital, was 103 (0.87%). Of the 78 patients with neonatal HIE, 11 did not pass the hearing screening. Therefore, the failure rate among patients with neonatal HIE was significantly higher (14.1% vs. 0.87%; *p* < 0.001) than that of all the newborns tested. The presence of HIs in the 11 patients who failed the hearing screening was further confirmed by ABR, OAE, and SSEP, and 17 individual ears exhibited hearing loss, of which 8 ears had mild (>35 and  ≤45 dB nHL), 0 ears had moderate (>45 and  ≤65 dB nHL), 1 ear had severe (>65 and  ≤90 dB nHL), and 8 ears had profound (>90 dB nHL) hearing loss. 

### 3.2. Demographic Data in Newborns with HIEs

Of the 78 patients with neonatal HIE, 32 patients were in stage I (mild), 30 were in stage II (moderate), and 16 were in stage III (severe). Of the stage 1 patients with neonatal HIE, 1 (3.1%), with mild HIE, had an HI in 1 ear, and 10 (21.7%) out of 46 patients with moderate or severe HIE, who were receiving hypothermia therapy, had HIs. 

### 3.3. Risk Factor of Hearing Damage in Patients with Mild and Moderate-to-Severe Neonatal HIE 

A higher rate of hearing loss among the patients with moderate-to-severe neonatal HIE was observed (χ2 (1, n = 78) = 5.39, *p* = 0.020; OR, 8.61; 75% CI 1.04 to 71.10). The demographic data of patients with neonatal HIE, with and without a hearing impairment, are presented in Table 1. Among 78 patients with neonatal HIE, differences in birth weight, sex, and age, an Apgar score of 1 min and 5 min, and method of delivery were nonsignificant between infants with HIs and those without HIs (Table 1). The lactic acid level was significantly higher in the patients with neonatal HIE and HIs than those without HIs (71.4 ± 48.4 vs. 104.8 ± 51.0; U = 181, *p* = 0.032; Table 2); the serum glucose level was also significantly higher in the patients with neonatal HIE and HIs than those without HIs (159.5 ± 86.1 vs. 112.1 ± 62.3; U = 166, *p* = 0.036; Table 2). Other factors, namely, white blood cell count (WBC) and the levels of platelets, hemoglobin, GOT, GPT, BUN, creatinine, lactate, LDH, troponin I, PT, aPTT, albumin, glucose, CK, CK-MB, K, and Na, did not differ significantly between the patients with HIs and those without HIs.

### 3.4. Hearing and Nonhearing Damage in Patients with Neonatal HIE Receiving Hypothermia Therapy 

A further analysis among 46 patients with moderate-to-severe HIE, who were receiving hypothermia therapy, revealed that those with and without HIs did not differ significantly in terms of their WBCs and levels of platelets, hemoglobin, GOT, GPT, BUN, creatinine, lactate, LDH, troponin I, PT, aPTT, albumin, glucose, CK, CK-MB, K, and Na. However, significance was determined among more patients who had HIs in clinical stage III (severe) (7 of 16, 43.8%; χ2 (1, *n* = 46) = 6.9862, *p* = 0.008) than those with HIs in clinical stage II (3 of 30, 10%; Table 3). 

### 3.5. MRI Findings in 11 Patients with HI 

**Figure 2:**
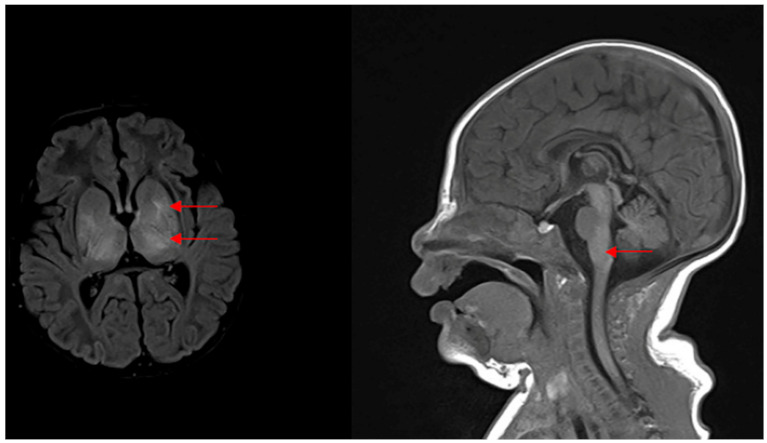
The brain magnetic resonance imaging (MRI) in P9 (Table 4) exhibited bilateral abnormalities of the basal ganglia, thalamus, and brainstem, which are involved in the nucleus of vestibulocochlear nerve with clinical stage III hypoxic–ischemic encephalopathy and profound hearing impairments.

Among 11 patients with neonatal HIE and HIs, 9 had abnormal brain MRI results and 2 had unremarkable brain MRIs (Table 4). The MRI of one patient with mild, stage I hearing loss indicated congenital cerebral hypoplasia, suggesting a congenital factor in the patient. Another five patients had mild hearing loss, two had unremarkable MRIs, and three had basal ganglion, thalamus, and midbrain MRI lesions. Diffuse white matter injury was noted in the brain of one patient with HIE and moderate hearing. Among five patients with severe-to-profound hearing loss, three cases were associated with symmetrical thalamus, basal ganglion, midbrain, and brainstem anomalies (Figure 2); one was associated with a subdural hematoma; one had diffuse white matter injury (Table 4). Overall, the degree of hearing loss was reflected in the degree of brain MRI anomalies, except for one profound case of hearing loss without brain parenchymal lesions.

The follow-up of patients with HIs at 1 year of age revealed the following neurodevelopmental outcomes: one was unremarkable, two had mild delays, five had moderate delays, and three had severe delays. One patient with mild HIE was intubated because of lung parenchymal disease, and exhibited nystagmus and motor delays. The degree of the patients’ HIs corresponded to their degree of neurodevelopmental delay at 1 year of age. 

## 4. Discussion

In our study, the clinical staging, and blood lactate and glucose levels measured within 6 h of birth were significantly related to the occurrence of HIs in patients with neonatal HIE. Selective neuron neurosis is a probable cause of HI because HIE may cause nuclei injury in the highly susceptible brainstem, as was observed in the brain MRIs of patients with HIE and profound HI. In our previous study [22], WBCs, and lactate, creatinine and LDH levels were revealed to be helpful biomarkers for initializing hypothermia therapy. The LDH level is a useful predictor because it indicates the level of cell necrosis. In our cases, the patients with profound HIs did not have high LDH levels, likely because of the nuclei injury in the brainstem. The early prediction of HI among patients with neonatal HIE, through rational, feasible biomarkers, is notable because it can lead to initializing early hearing habilitation programs for those neonates. 

We noted that the HIs in patients with neonatal HIE were often combined with other neurological irregularities, including feeding problems, facial diplegia, severe cerebral palsy, or midbrain or brainstem lesions. Brain MRIs highlighted the cranial nuclei and midbrain lesions in patients with selective neuronal necrosis as their HIE progressed. In the study, the neonatal hearing defect in the hearing screenings of all the newborns was approximately 8–9/1000, and was higher than the approximately 3–4/1000 infants [23,24]. This difference may be due to the selected population. In our cohort, the majority of hearing impairments were in those patients admitted to a neonatal intensive care unit. The patients with neonatal HIE had a higher risk of HI than newborns among the general population.

Few studies have reported on biomarkers correlated with HI in neonatal HIE [25,26,27]. In our study, glucose and lactate are risk factors for HI in neonatal HIE, not including liver function, which was reported in one study [26], probably due to the selected cases of patients with neonatal HIE and severe shock injury. Liver function impairment is likely due to ischemic injury. The ability of lactate to predict severe MRI lesions that typically correlate with poor neurodevelopmental outcomes and HI among patients at 1 year of age is valuable [22]. Other biomarkers, namely, lactate, glucose, PT, aPTT, albumin, and creatinine, also help to predict HIE severity. However, in this study, lactate, aPTT, and albumin levels were not found to significantly contribute to hearing loss in the hypothermia group. One possible reason for this is that therapeutic hypothermia can rescue brain injury and hearing injuries in neonatal stage II–III HIE. Therapeutic hypothermia likely targets multiple mechanisms, contributing to otoprotection, including slowed metabolism and reduced oxidative stress [28]. However, further case studies are warranted. We observed that clinical staging was the strongest clinical biomarker in the hypothermia group. A critical sign of stage III HIE is a lack of crying because it may highlight impairments in the pathway from the brainstem to the thalamus to the cortex, and an injury of the seventh cranial nerve nucleus that is close to the eighth (vestibulocochlear) cranial nerve nucleus. Therefore, clinicians should examine patients for additional clinical signs and biomarkers.

Our findings indicate that the cause of HI in neonatal HIE may be the susceptibility of the patients to eighth nucleus injury during hypoxic change. This is reflected in the profound hearing loss, and midbrain and brainstem lesions. The degree of HI is also correlated with the degree of brain injury. The early identification of HI in patients with neonatal HIE is critical to their long-term neurodevelopmental outcomes. Early MRI is strongly correlated with later neurodevelopmental outcomes, which, particularly in the thalamus and basal ganglion, was often associated with HI in our study. Isolated cyst encephalomalacia that does not involve the midbrain and brainstem causes less HI. One hypothesis is that HI is related to hypothermia therapy; however, we found that HI was associated with the degree of brain injury. Hypothermia therapy might diminish HI among patients with HIE, despite no literature having reported this finding. Early hypothermia therapy for patients with moderate-to-severe HIE is critical to the brain, and to saving hearing functions.

This study has some limitations. We presented a limited number of cases of HIE and HI in the analysis of the risk factors; hence, our findings might be biased. The data were compared with those of more than 10,000 newborns without HIE, who had a background of congenital hearing loss. Therefore, studies including a larger number of patients with neonatal HIE and HI are warranted. 

## 5. Conclusions 

Clinical staging, and early blood lactate and blood glucose levels, within 6 h after birth, can be predicting factors for HIs among patients with neonatal HIE. Among patients with clinical moderate-to-severe HIE, only clinical staging was a predicting factor. The severity of the HI reflected the degree of injury to the vestibulocochlear nerve nuclei in the brain. The early identification of HI in patients with neonatal HIE is critical for developing timely interventions for children’s hearing habilitation.

## Figures and Tables

**Table 1:** Demographic data of 78 newborns with neonatal hypoxic–ischemic encephalopathy (HIE) with and without hearing impairment (HI).

	Neonatal HIE without Hearing Impairments (N = 67)	Neonatal HIE with Hearing Impairments (*N* = 11)	*p* Values
Gestational age (weeks)	38.6 ± 1.2	38.7 ± 1.5	t(76) = −0.213, *p* = 0.835
Birth weight (gm)	2989.2 ± 459.3	2928.3 ± 472.1	t(76) = 0.389, *p* = 0.699
Gender			
Male	41 (61.2%)	6 (54.5%)	χ2 (1, *n* = 77) = 0.174, *p* = 0.676
Female	26 (38.8%)	5 (45.5%)	
Method of delivery			
Cesarean section	24 (35.8%)	4 (36.4%)	χ2 (1, *n* = 77) = 0.001, *p* = 0. 972
aginal delivery	43 (64.2%)	7 (63.6%)	
Transfer mode			
Inborn	27 (40.3%)	3 (37.5%)	χ2 (1, *n* = 77) = 0.677, *p* = 0. 411
Outborn	40 (59.7%)	8 (62.5%)	
Apgar score at one minute	3.1 ± 0.6	2.6 ± 1.0	t(77) = 1.481, *p* = 0.144
Apgar score at five minutes	6.4 ± 0.9	6.1 ± 1.0	t(77) = 1.042, *p* = 0.303

HIE, hypoxic–ischemic encephalopathy.

**Table 2:** Biomarkers of 78 patients with neonatal HIE with and without HI.

Biomarkers	Neonatal HIE without Hearing Impairments (*N* = 67)	Neonatal HIE with Hearing Impairments (*N* = 11)	*p* Values
WBCs (9100–34,000 mm 3 µL)	20,096.0 ± 7969.2	26,223.6 ± 14,562.1	U = 219, *p* = 0.101
Platelet (84–478 mm 3 µL)	230,258.7 ± 73,565.2	237,181.8 ± 83,798.4	t(76) = −2.79, *p* = 0.781
Hemoglobin (13.88 ± 1.34 g/dL)	17.4 ± 6.2	16.7 ± 1.3	t(76) = 0.42, *p* = 0.678
SGOT (30–100 U/L)	175.5 ± 257.9	194.0 ± 329.1	U = 270, *p* = 0.695
SGPT (6–40 U/L)	53.3 ± 91.9	52.1 ± 64.9	U = 239, *p* = 0.306
BUN (3–12 mg/dL)	10.5 ± 3.2	13.9 ± 7.1	U = 219, *p* = 0.149
Creatinine (0.03–0.50 mg/dL)	0.9 ± 0.2	1.1± 0.3	U = 244, *p* = 0.354
Lactate * (4.4 to 14.4 mg/dL)	**71.4 ± 48.4**	**104.8 ± 51.0**	**U = 181, *p* = 0.032**
LDH (170–580 U/L)	1207.3 ± 1341.2	1398.2 ± 2127.8	U = 300, *p* = 0.976
PT (13.0 ± 1.43 s)	15.9 ± 4.4	22.5 ± 15.1	U = 249, *p* = 0.314
aPTT (42.9 ± 5.80 s)	60.3 ± 21.1	72.1 ± 30.7	U = 228, *p* = 0.393
Albumin (2.5–3.4 g/dL)	3.6 ± 0.4	3.4 ± 0.5	t(76) = 0.995, *p* = 0.781
Glucose * (40–60 mg/dL)	**112.1 ± 62.3**	**159.5 ± 86.1**	**U = 166, *p* = 0.036**
Na (133–146 mmol/L)	135.8 ± 3.6	135.4 ± 2.9	t(76) = 0.421, *p* = 0.675
K (3.2–5.5 mmol/L)	4.1 ± 0.7	4.1 ± 0.6	t(76) = 0.222, *p* = 0.825
CK (39–308 U/L)	2795.8 ± 3979.7	974.7 ± 830.4	U = 187, *p* = 0.125
CK-MB (0–4.5 ng/mL)	60.9 ± 86.9	58.6 ± 56.1	U = 82, *p* = 0.822
Troponin I (0–30 pg/mL)	217.8 ± 626.8	321.1 ± 564.1	U = 161, *p* = 0.197

* Bold fonts indicate significance; * *p* < 0.05. HIE, hypoxic–ischemic encephalopathy; ST, standard deviation; WBCs, white blood cells; GOT, aspartate transaminase; GPT, alanine transaminase; BUN, blood urea nitrogen; LDH, lactate dehydrogenase; PT, prothrombin time; aPTT, activated partial thromboplastin time; CK, creatine phosphokinase; CK-MB, creatine kinase Mb; K, potassium; Na, sodium.

**Table 3:** Biomarkers in patients with neonatal HIE with and without HI who were receiving hypothermia therapy.

Clinical Staging and Biomarkers	Neonatal HIE with Hypothermia with Hearing Impairments (N = 10)	Neonatal HIE with Hypothermia without Hearing Impairments (N = 36)	*p* Values
**Clinical stage II (N = 30)**	**3 (10.0%)**	**27 (9.00%)**	**χ2 (1, *n* = 46) = 6.9862, *p* = 0.008**
**Clinical stage III (N = 16)**	**7 (43.7%)**	**9 (56.3%)**	
WBCs (9100–34,000 mm 3 µL)	27,584.0 ± 14,584.5	22,146.1 ± 8871.9	U = 127, *p* = 0.228
Platelet (84–478 mm 3 µL)	232,100.0 ± 87,601.9	223,882.4 ± 68,001.7	t(44) = −0.314, *p* = 0.755
Hemoglobin (13.88 ± 1.34 g/dL)	16.8 ± 1.3	17.9 ± 7.8	t(44) = 0.445, *p* = 0.659
SGOT (30–100 U/L)	209.2 ± 342.8	230.7 ± 308.1	U = 149, *p* = 0.556
SGPT (6–40 U/L)	56.2 ± 67.0	73.4 ± 111.1	U = 150, *p* = 0.585
BUN (3–12 mg/dL)	14.4 ± 7.4	11.0 ± 3.3	U = 120, *p* = 0.155
Creatinine (0.03–0.50 mg/dL)	1.1 ± 0.3	1.0 ± 0.2	U = 148, *p* = 0.538
Lactate (4.4 to 14.4 mg/dL)	87.0 ± 52.7	111.1 ± 49.0	U = 114, *p* = 0.117
LDH (170–580 U/L)	1501.3 ± 2213.7	1557.1 ± 1619.6	U = 147, *p* = 0.605
Troponin I (0–30 pg/mL)	358.2 ± 591.3	314.6 ± 775.5	U = 97, *p* = 0.347
PT (13.0 ± 1.43 s)	23.5 ± 15.7	16.8 ± 4.8	U = 142, *p* = 0.425
aPTT (42.9 ± 5.80 s)	74.4 ± 31.6	64.1 ± 22.3	U = 129, *p* = 0.540
Albumin (2.5–3.4 g/dL)	3.39 ± 0.50	3.52 ± 0.50	t(44) = 0.725, *p* = 0.473
Glucose (40–60 mg/dL)	122.1 ± 76.8	164.2 ± 90.0	U = 103, *p* = 0.163
Na (133–146 mmol/L)	135.6 ± 3.0	135.6 ± 3.8	t(44) = 0.036, *p* = 0.971
K (3.2–5.5 mmol/L)	4.0 ± 0.7	4.2 ± 0.8	t(44) = 0.405, *p* = 0.688
CK (39–308 U/L)	1018.3 ± 868.3	3689.1 ± 4767.0	U = 103, *p* = 0.136
CK-MB (0–4.5 ng/mL)	69.7 ± 58.2	74.2 ± 106.0	U = 38, *p* = 0.670

Bold fonts indicate *p* < 0.05; HIE, hypoxic–ischemic encephalopathy; ST, standard deviation; WBCs, white blood cells; GOT, aspartate transaminase; GPT, alanine transaminase; BUN, blood urea nitrogen; LDH, lactate dehydrogenase; PT, prothrombin time; aPTT, activated partial thromboplastin time; CK, creatine phosphokinase; CK-MB, creatine kinase Mb; K, potassium; Na, sodium.

**Table 4:** Brain MRI results in patients with neonatal HIE with HI.

Patient Number/Brain MRI Lesions	Sarnat Staging	Hearing Loss (Left Side)	Hearing Loss (Right Side)	Thalamus or Basal Ganglion	Brainstem (One of Midbrain, Pons, and Medulla)	Multicystic Encephalomalacia	Diffuse White Matter Injury	SDH	Others	Unremarkable
**P1**	1	Normal	Mild						Cerebellar hypoplasia	
**P2**	2	Mild	Normal							+
**P3**	3	Mild	Mild							+
**P4**	3	Mild	Mild	+	+					
**P5**	3	Normal	Mild	+	+					
**P6**	3	Mild	Normal	+	+					
**P7**	2	Severe	Normal				+	+		
**P8**	2	Profound	Profound					+		
**P9**	3	Profound	Profound	+	+					
**P10**	3	Profound	Profound	+	+	+	+	+		
**P11**	3	Profound	Profound	+	+	+	+	+		

+ indicates lesions on MRI; MRI, magnetic resonance imaging; SDH, subdural hemorrhage.

## Data Availability

The datasets used and/or analyzed during the current study are available from the corresponding author on reasonable request.
